# Multiple dermoid sinuses of type Vb and IIIb on the head of a Saint Bernard dog

**DOI:** 10.1186/1751-0147-55-62

**Published:** 2013-09-05

**Authors:** Anna Perazzi, Michele Berlanda, Massimo Bucci, Silvia Ferro, Roberta Rasotto, Roberto Busetto, Ilaria Iacopetti

**Affiliations:** 1 Department of Animal Medicine, Production and Health, University of Padova, Padova, Italy; 2 Department of Comparative Biomedicine and Food Science, University of Padova, Padova, Italy

**Keywords:** Dermoid sinus, Fronto-occipital region, Saint Bernard dog

## Abstract

Dermoid sinus, a congenital malformation of neural tube development, has been reported in humans and several animal species including dogs. It is typically found in the dorsal midline and commonly occurs in the Rhodesian Ridgeback breed. A case of multiple dermoid sinuses in the fronto-occipital region is described. An 11-month-old, intact female Saint Bernard dog was presented with a 2 day history of discharge from a large irregular subcutaneous mass in the fronto-occipital region. The dog was otherwise healthy. The dog had two circular skin lesions (approximately 4 × 4 and 4 × 2 cm diameter) surrounded by multiple irregular elevated masses. The masses had multiple small openings on the skin surface with tufts of hair protruding from the apertures. The masses were surgically removed, and the diagnosis of multiple dermoid sinuses was confirmed by histological examination. Histopathological examination showed multiple, variably sized, spherical to tubular cysts expanding the dermis and subcutis. Cysts were filled with hair shafts and lamellar keratin and were lined by a stratified squamous epithelium. Sebaceous and apocrine gland adnexal structures were also observed. To the best of our knowledge, this is the first reported case of multiple dermoid sinuses of two different types in the head of a Saint Bernard dog.

## Background

Dermoid sinus (DS) is a congenital malformation caused by an incomplete separation of the skin and neural tube during embryonic development [1]. This abnormality occurs in humans [2-8], dogs [9-40], cats [32,41-44], horses [45], cattle [46], goats [47], sheep [48] buffalo [49] and camels [50]. A review of canine cases is presented in Table 1. Cutaneous DS has been well-documented in Rhodesian and Thai Ridgeback dogs and their cross breeds [51]. DS is thought to be inherited in Rhodesian Ridgebacks. Some studies concluded that the ridge is an autosomal dominant trait that predisposes for DS [51,52]. Isolated cases of DS have also been reported in other breeds (Table 1) [9-40], without evidence of a genetic predisposition [34,51]. Several authors have reported that DSes are found most frequently in the cervical and thoracic regions [11,14,38] with possible extension to the meninges and subarachnoid space [9,21,23,32,33,35-37,39,40]. They are less frequently found in the sacral region [38]. Neurological signs may be present if there is communication with the dura mater and subsequent inflammation of the spinal cord [16]. DSes in dogs have also been reported to occur only on the parieto-occipital region of the cranium [14] and on the nose [9-12]. This case report describes an unusual combination of multiple DSes of two different types on the head (fronto-occipital region) of a Saint Bernard dog.

**Table 1:** Clinical cases of canine dermoid sinus reported in the veterinary literature

**Location**	**Breed affected**	**Type and subtype**	**References**
Nose	American Cocker Spaniel	IVc°	[9]
	American Cocker Spaniel	Ic°	[10]
	Brittany Spaniel	Ic°	[10]
	Dalmatian	Vc*	[11]
	English Bull Terrier	Ic°	[12]
	Golden Retriever	Ic°	[10]
	Shih-tzu	Ic°	[13]
	Springer Spaniel	Ic°	[10]
Head	Rottweiler	IIb and IIIb*	[14]
Cervical region	Borboel	IIa°	[15]
	Chow-Chow	IIa and IIIa°	[16]
	Golden Retriever	IIa°	[17]
	Great Pyrenees dog	IIIa°	[18]
	Rhodesian Ridgeback	Ia, IIa and IIIa°	[19]
	Rhodesian Ridgeback	IIa°	[20]
	Rhodesian Ridgeback	Iva°	[21]
	Rhodesian Ridgeback	IIa°	[22]
	Rhodesian Ridgeback	Iva°	[23]
	Rhodesian Ridgeback	VIa°	[24]
	Rhodesian Ridgeback	IIa°	[25]
	Rhodesian Ridgeback	Va*	[26]
	Rhodesian Ridgeback	Ia°	[27]
	Rhodesian Ridgeback	IIa°	[28]
	Rhodesian Ridgeback	IIa*	[29]
	Rhodesian Ridgeback	Ia°	[30]
Thoracic region	Boxer	IVa°	[31]
	Chinese Crested dog	IVa*	[32]
	Chow-Chow	IIIa and IIa°	[16]
	Rhodesian Ridgeback	Ia, IIa and IIIa°	[19]
	Shih-tzu	IVa°	[31]
	Shih-tzu	IVa*	[33]
	Siberian Husky	IIa°	[34]
	Swedish Vallhunds	IVa*	[32]
	Swedish Vallhunds	VIa*	[32]
	Victorian Bulldog	IVa*	[35]
	Yorkshire Terrier	IVa°	[36]
	Yorkshire Terrier	IVa*	[37]
Lumbosacral region	English Springer Spaniel	VIa°	[38]
Sacrococcygeal region	Rhodesian Ridgeback	Iva°	[39]
	Rhodesian Ridgeback	Ia, IIa and IIIa°	[19]
	Rhodesian Ridgeback	IVa°	[40]

Reported cases of dermoid sinus with anatomical location, breed and type and classification [* = stated by the authors; ° = deduced from the text according with the classifications of Kiwiranta [32] and Bornard [14]].

## Case presentation

**Figure 1:**
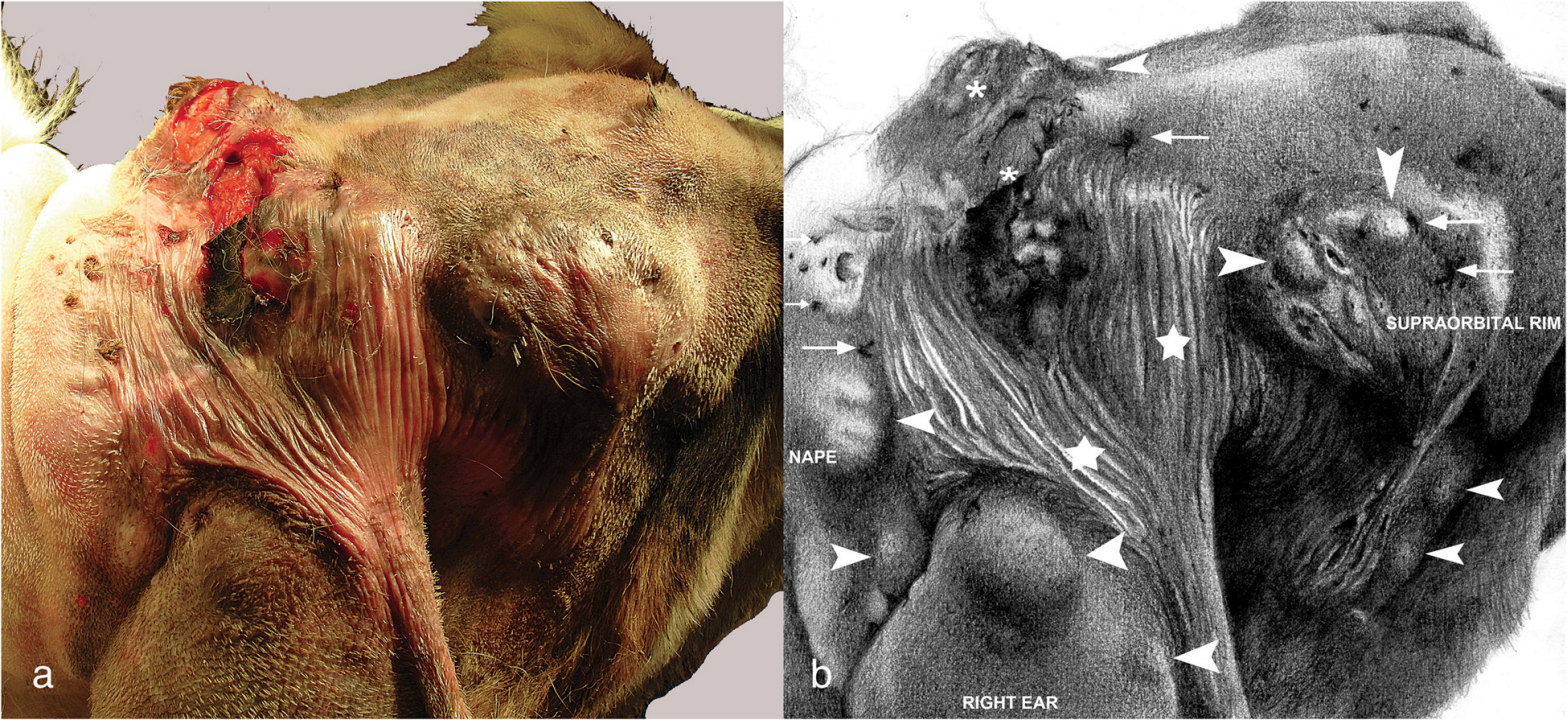
**Photograph and illustration of fronto-occipital region of the dog. a)** Dorso-lateral view of head of dog (mouth on the right side, neck on the left side). The lesions involve an area between the right supraorbital rim, the base of the right ear and nape, extending partially into the fronto-occipital area on the left. **b)** Illustration of the same photograph that better shows the distribution and the tridimensional characters of the nodular lesions. In the center are two big circular skin lesions (*asterisks*) draining serous and purulent material, surrounded by multiple irregular swollen masses (*arrow heads*) and multiple small openings on the skin surface with tufts of hair protruding from the skin apertures (*arrows*). There is an extensive area around and under the ulceration characterized by thin and translucent glabrous epidermis (stars).

An 11-month-old, 42 kg, intact female Saint Bernard dog was presented with a 2 day history of discharge from a large, irregular swelling in the fronto-occipital region. The owner reported that the dog had scratched the head on trees almost daily since it was a puppy. Palpation of the head elicited a marked pain reaction, but no other clinical signs were observed. The discharge was submitted for bacterial culture and antimicrobial sensitivity test, and a growth of coagulase positive staphylococci was obtained. Routine haematology revealed only a mild leucocytosis (17.5 × 10^3^/μL, normal range 5 to 13 × 10^3^/μL) and biochemistry was unremarkable. Hair was clipped from the area, revealing abnormal skin which covered the area between the right supraorbital rim, the base of the right ear and nape, extending partially into the left fronto-occipital portion of the skull (Figure 1a and 1b). Two large circular skin lesions of approximately 4 × 4 and 4 × 2 cm diameter with serous and purulent exudate were located in the central part of this area. These lesions were surrounded by multiple irregular swollen masses containing multiple small openings on the skin surface with tufts of hair protruding from the apertures. The region overlying the right ear was characterized by a thin and hairless skin (Figure 1a and 1b). A tentative diagnosis of multiple cutaneous DSes was made despite the atypical location, breed and the large number of lesions. The dog was treated with amoxicillin-clavulanic acid (Synulox, Pfizer A.H., New York, USA) at a dose of 20 mg/kg twice daily *per os*, combined with enrofloxacin (Baytril, Bayer, Leverkusen, Germany) at a dose of 5 mg/kg once daily *per os* starting 3 days before surgical removal of the abnormal tissue. General anaesthesia with the dog positioned in ventral recumbency were induced and the area aseptically prepared and draped. An incision allowing wide exposure of the tissue and blunt dissection was made. After complete excision of the lesion, two Penrose drains were placed and the wound was sutured. A small part of the incision overlying the right ear had to be left to heal by secondary intention to avoid excessive traction of sutures. Postoperative analgesia and antibiotic treatment were administered and a light bandage was applied for the first two weeks.

**Figure 2:**
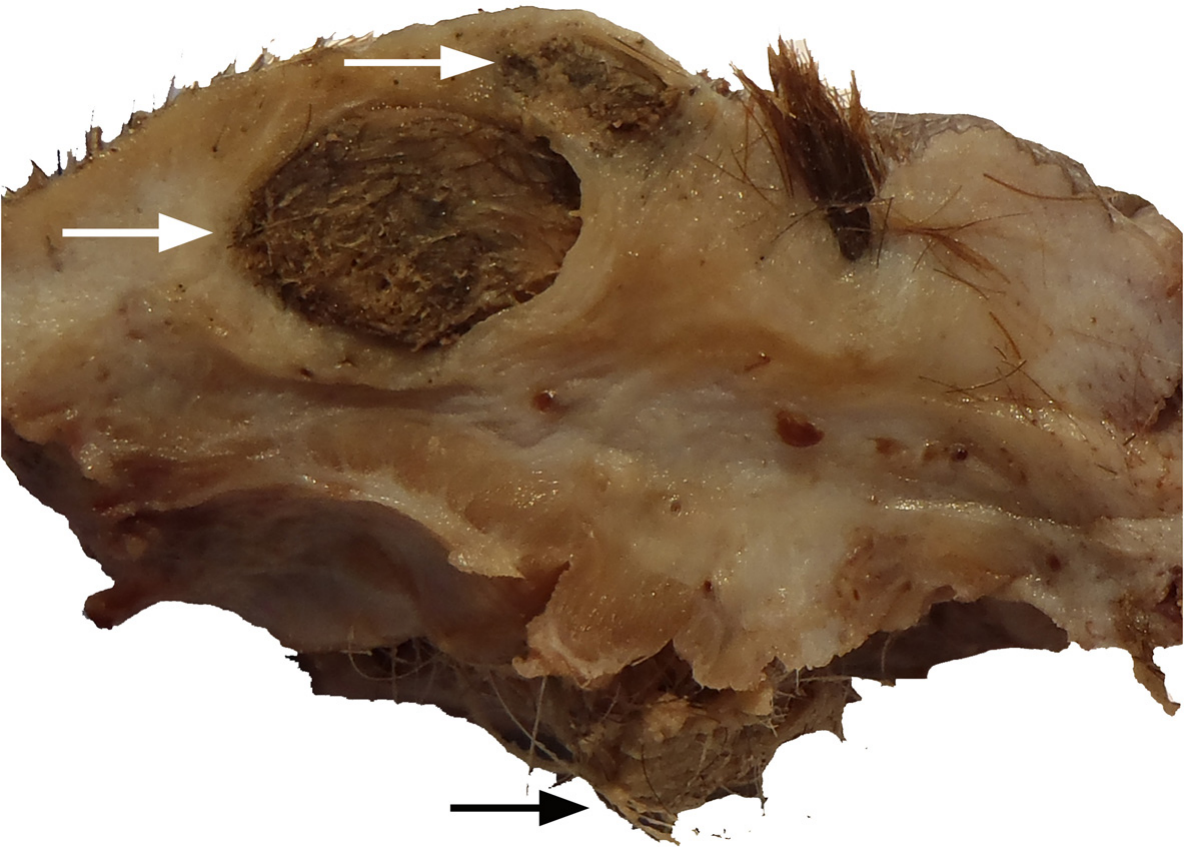
**Macroscopic aspect of the cystic skin at cut surface.** Formalin fixed specimen. White arrows indicate two subcutaneous cysts. At the right of the cysts a tuft of hair protruding from a sinus toward the surface is evident. Ectopic hairs are evident (black arrow) in the subcutaneous tissue. Skin and subcutis, dog.

The excised tissue appeared on cut section to be formed by multiple adjacent dermal and subcutaneous cysts with no apparent connections (Figure 2). Cysts were filled with hair, keratin, and waxy purulent fluid. The overlying skin had several epidermal invaginations with a dense tuft of hairs frequently protruding from them. Resected tissue was processed for routine histopathology.

**Figure 3:**
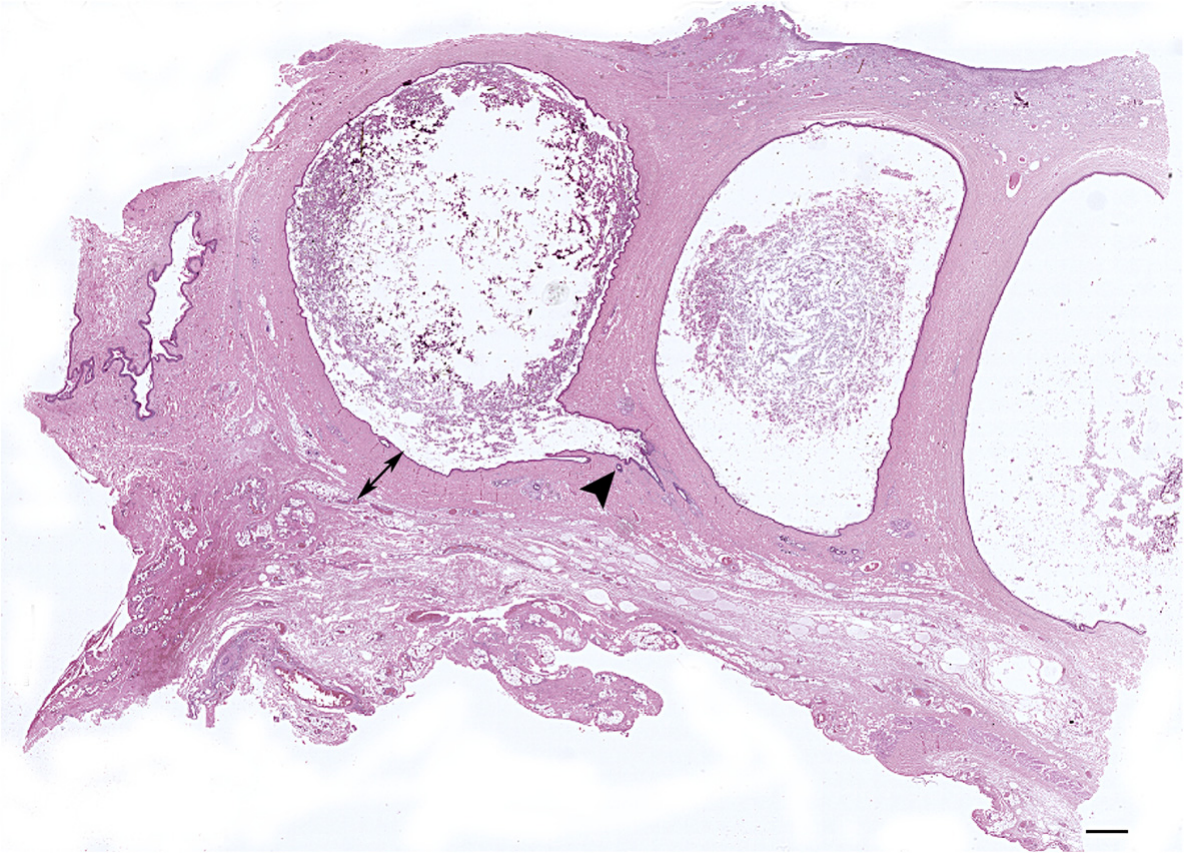
**Microphotograph of adjacent dermoid cysts.** The figure shows multiple subcutaneous cysts embedded in underling dermis (double headed arrow). The arrow head point at a follicular unit opening in the cyst. The overlying skin is ulcerated with no adnexa. Skin and subcutis, dog. Bar = 1000 μm, haematoxylin and eosin stain.

**Figure 4:**
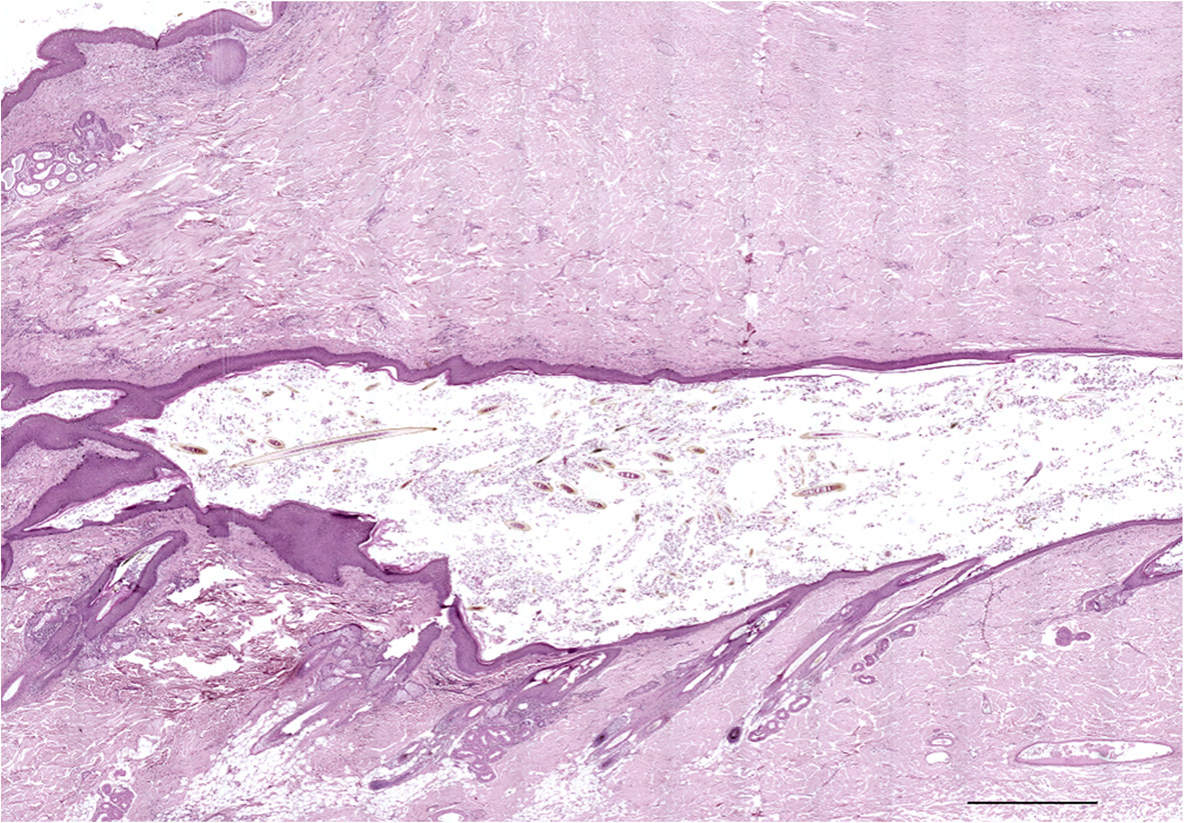
**Histological characteristics of the wall cyst.** A portion of a cyst with keratin and several hair shafts in the lumen. The cyst wall at the bottom of the figures contains numerous radial folliculosebaceous units that open in to the cyst. The dermis over the cyst is fibrotic without hair follicles. On the upper left corner is evident a small portion of the wall of another cyst. Deep dermis and subcutis, dog. Bar = 1000 μm, haematoxylin and eosin stain.

**Figure 5:**
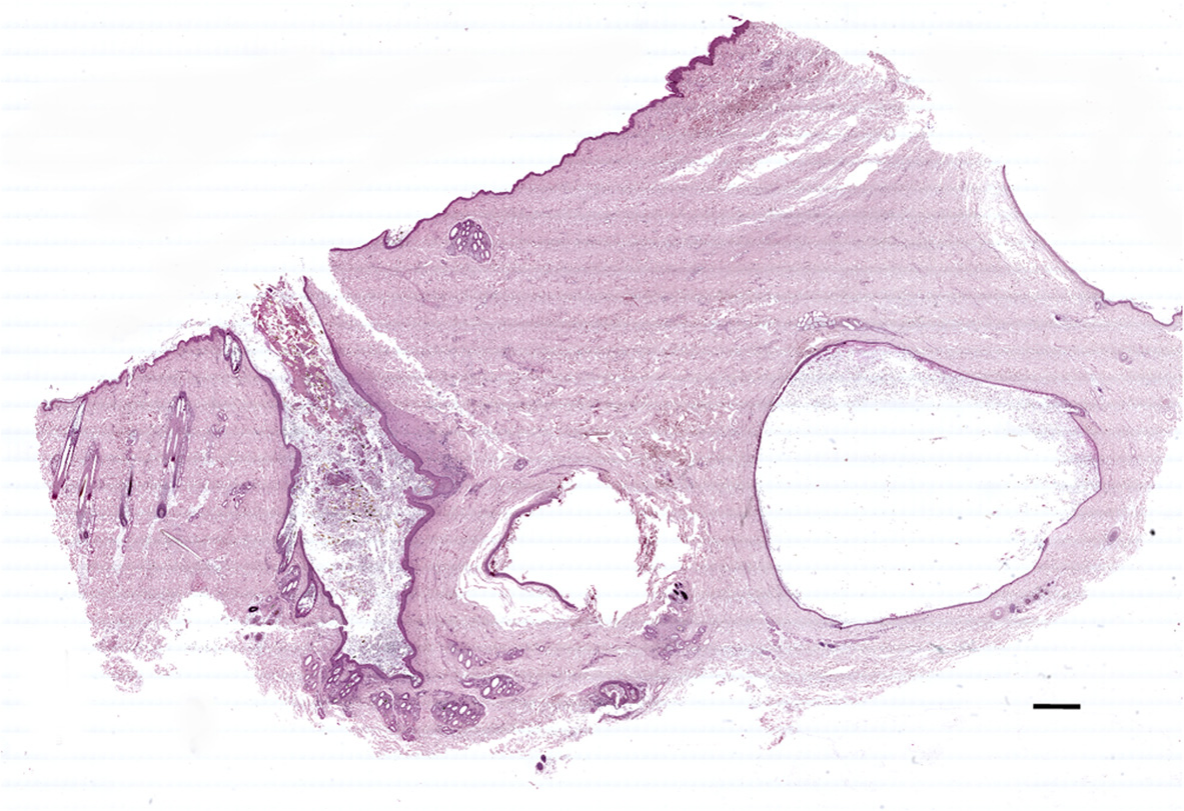
**Microphotograph of a dermoid sinus.** The figure shows on the left an open sinus surrounded by follicular units, and on the right two deep cysts. The dermis above the cysts contains few or no adnexa, and the epidermis is thin with an abrupt transition to hyperplastic epidermis on the right of the section. Skin and sub-cutis, dog. Bar = 1000 μm, haematoxylin and eosin stain.

Multiple, variably sized, spherical to tubular cysts expanding the dermis and subcutis were seen histologically. Cysts were filled with hair shafts and lamellar keratin and were lined by a stratified squamous epithelium with the granular layer occasionally evident (Figure 3). Cysts were surrounded by a thin rim of collagen bundles that tended to run parallel to the cyst walls (Figure 4). Within this collagen, multiple folliculosebaceous units radiated perpendicularly from the cyst walls. Occasionally, cysts communicated with the overlying epidermis via a pore (Figure 5). The surrounding epidermis was slightly compressed, atrophic, and multifocally ulcerated. The dermis at the periphery showed moderate fibrosis, dislocation of adnexa, and moderate multifocal granulomatous inflammation with a few foreign body type multinucleated giant cells surrounding keratin debris. A final histopathologic diagnosis of multiple DSes was made.

At a 6-month follow-up, the patient appeared to be in good health and the owner reported no further problems with head scratching or skin sensitivity.

## Discussion and conclusions

The term DS is generally considered to be synonymous with dermoid cyst and pilonidal cyst [16,23,26,53-55]. However, some authors recently proposed that a distinction should be made in dogs and cats, as it is done in humans, between dermoid cysts and DSes based on the presence in the latter of a connection to the skin [4,27,56,57]. Similarly, other authors identified different types of DS in dogs and considered dermoid cyst as a specific type of DS lacking an opening on the skin [19,26,35,55]. Based on the extent of penetration into the subcutaneous tissue, four types of DSes were initially recognized in veterinary medicine: type I extends ventrally as a cylindrical sac attached to the supraspinous ligament, type II consists of a sac-like portion that is more superficial than that of type I and is attached to the ligament by a fibrous band, type III is made up of a superficial sac with no attachment to the supraspinous ligament and type IV extends to the spinal canal and is attached to the dura mater [19]. Later, two other types of DS, type V [16] and type VI [32], were introduced. Type V was described as a true dermoid cyst consisting of a closed epithelial-lined sac difficult to detect via palpation of the skin [57]. This can be considered a more accurate use of the term ‘dermoid cyst’ since fistulous tract formation or connection to the epidermis is absent. In type VI the open sinus tract reaches the level of the supraspinous ligament and connects via a fibrous cord without a lumen to the dura mater [35]. Based on the anatomical location, all these types of DS were recently further classified in three subtypes: subtype “a” (dorsal midline), subtype “b” (head, excluding nose), and subtype “c” (nose) [14]. DSes described in veterinary literature according to classifications of Kiwiranta [32] and Bornard [14], their localization, and affected breeds are summarized in Table 1[9-40].

Based on the multiplicity of the lesions and atypical localization, we classified the present case as a combination of DSes type Vb and type IIIb. To the best of our knowledge, this is the first report of a case of different types of DS on the head in a Saint Bernard dog. In the veterinary literature, there are only two other studies that described true cutaneous dermoid cysts (DS type V) in dogs [11,26] and neither of them were of subtype b. Our case is also unusual for its presentation on the head, for the breed affected and the multiplicity of the lesions, a pattern rarely described in dogs or humans [6,14,16,19]. DS is reported as a congenital or acquired lesion [3,6,41]. Considering that the dog described here was 11-months-old, a congenital disorder was suspected. Even if DSes are usually diagnosed at birth, in some cases they are asymptomatic initially and discovered later in life when they become distended or infected [41]. The owner in this case complained about the swelling only when the discharge became visible, and the external openings were seen only after shaving the area.

Histopathology was necessary to confirm the clinical impression [58]. The most important differential diagnoses were follicular infundibular cyst, folliculosebaceaous hamartoma and trichofolliculoma [53]. Folliculosebaceous hamartoma was ruled out because randomly distributed sebaceous lobules are usually more evident. In a trichofolliculoma, the epithelium lining the cyst should have some signs of isthmus/matrical differentiation that was lacking in our case. Distinction from a follicular infundibular cyst can sometimes be problematic. In this case a DS was diagnosed because the folliculosebaceous units radiating from the cyst wall were oriented perpendicular to it, while hair follicles surrounding follicular infundibular cysts usually maintain the normal perpendicular orientation to the epidermis. In addition, the concentric arrangement of the surrounding collagen, well evident in our case, is typical of DS and not present in infundibular cysts [53]. In conclusion, this appears to be the first report of multiple DSes types IIIb and Vb in a young Saint Bernard dog.

## Competing interests

The authors declare that they have no competing interests.

## Authors’ contributions

AP and MBe conceived of the study and participated in its design and coordination and helped to draft the manuscript. MBu did the clinical investigation. SF and RR performed the histopathologic examination and interpretation. II made an intellectual contribution and reviewed the paper. RB has given final approval of the version to be published. All authors read and approved the final manuscript.
